# Asymmetrical cortical vein sign predicts early neurological deterioration in acute ischemic stroke patients with severe intracranial arterial stenosis or occlusion

**DOI:** 10.1186/s12883-020-01907-w

**Published:** 2020-09-02

**Authors:** Wei Li, Wei-Min Xiao, Gen-Pei Luo, Yong-Lin Liu, Jian-Feng Qu, Xue-Wen Fang, Fang Wang, Yang-Kun Chen

**Affiliations:** 1 Department of Neurology, Affiliated Dongguan People’s Hospital, Southern Medical University, Wandao Road South No.3, Wanjiang District, Dongguan, Guangdong Province China; 2 Department of Radiology, Affiliated Dongguan People’s Hospital, Southern Medical University, Dongguan, Guangdong Province China

**Keywords:** Acute ischemic stroke, Asymmetrical prominent cortical vein sign, Intracranial arterial stenosis or occlusion, Susceptibility-weighted imaging, Hypoperfusion, Early neurological deterioration

## Abstract

**Background:**

Susceptibility weighted imaging (SWI) provides an approximate assessment of tissue perfusion and shows prominent hypointense cortical veins in the ischemic territory because of the increased concentration of deoxyhemoglobin. We aimed to evaluate whether asymmetrical prominent cortical vein sign (APCVS) on SWI can predict early neurological deterioration (END) in acute ischemic stroke patients with severe intracranial arterial stenosis or occlusion (SIASO).

**Methods:**

One hundred and nine acute ischemic stroke patients with SIASO who underwent SWI were retrospectively recruited. END was defined as an increase in the National Institutes of Health Stroke Scale score ≧2 points despite standard treatment in the first 72 h after admission. The APCVS was defined as more and/or large vessels with greater signal loss than those in the opposite hemisphere on SWI.

**Results:**

Thirty out of the 109 (27.5%) patients developed END. Sixty (55.0%) patients presented with APCVS on SWI. APCVS occurred in 24 (80%) patients with END, whereas it only occurred in 36 (45.6%) patients without END (*P* = 0.001). Patients with APCVS were more likely to have END (40.0%, vs. 12.2%, *P* = 0.001) than those without END. Multivariate logistic regression indicated that APCVS (OR = 4.349, 95% C.I. = 1.580–11.970, *P* = 0.004) was a significant predictor of END in acute ischemic stroke patients with SIASO, adjusted for previous stroke history and acute infarct volume.

**Conclusions:**

In acute ischemic stroke patients with SIASO, the APCVS might be a useful neuroimaging marker for predicting END, which suggests the importance of evaluation of perfusion status.

## Background

Stroke is the leading cause of death in China [1, 2]. Intracranial atherosclerosis is a frequent etiology of acute ischemic stroke (AIS) in the Chinese population, with a prevalence of 46.6% [3]. Patients with AIS caused by intracranial atherosclerosis stenosis or occlusion have a higher risk of early neurological deterioration (END), and the possible underlying mechanisms include hemodynamics, hypoperfusion, or thrombus extension [4–7].

Perfusion status in AIS patients is often evaluated using computed tomography (CT) perfusion or magnetic resonance perfusion, which require additional contrast. In recent years, susceptibility-weighted imaging (SWI) has been applied for the detection of hypoperfusion in AIS patients [8, 9]. This high-resolution three-dimensional echo magnetic resonance imaging (MRI) technique is highly sensitive to paramagnetic material such as deoxyhemoglobin and hemosiderin. The asymmetrically prominent cortical vein sign (APCVS) is typically identified as asymmetrical dilated-vessel-like signal loss seen in the cortex on SWI. It is thought to represent either a penumbra or poor collateralization of the arterial supply [10, 11]. The APCVS is a potential neuroimaging marker for the evaluation of hemodynamics in patients with moyamoya disease [12] and for predicting arterial occlusion in AIS patients [13]. According to Sharma et al. [14], internal cerebral vein asymmetry on follow-up CT angiography is an early predictor of poor functional outcome after intravenous thrombolysis. Another study suggested that the APCVS predicts the clinical course and outcome of anterior circulation ischemic stroke patients in the acute phase and the 90-day prognosis. A positive relationship was also observed between an APCVS and END [15]. However, in most cases, the APCVS occurs in patients with severe intracranial arterial stenosis or occlusion (SIASO), which indicates that SIASO should be considered in the analysis of AIS patients. It is unknown whether an APCVS increases the risk of END in AIS patients with SIASO. Therefore, the purpose of this study was to investigate the relationship between the APCVS and END in AIS patients with SIASO.

## Methods

### Participants and setting

AIS patients admitted to Dongguan People’s Hospital between July 1, 2016 and December 31, 2018 were screened retrospectively. The inclusion criteria were (1) age ≥ 18 years; (2) diagnosis of AIS of the anterior circulation and hospitalized within 24 h after onset; (3) symptomatic SIASO confirmed by magnetic resonance angiography (MRA). Symptomatic SIASO refers to ipsilateral artery stenosis or occlusion and was defined as more than 70% diameter loss in any segment of the internal carotid artery (ICA)/middle cerebral artery (MCA) ipsilateral to the infarction. Intracranial stenosis was assessed using the Warfarin Aspirin Symptomatic Intracranial Disease (WASID) Criteria [16]; (4) pre-stroke modified Rankin scale ≤1; and (5) an SWI examination was performed. The exclusion criteria included (1) patients without MRI within 24 h after admission or those who had poor-quality MRI information; (2) acute infarction involving the posterior circulation or bilateral hemisphere confirmed by diffusion-weighted imaging (DWI); (3) contralateral ICA or MCA stenosis (more than 50% diameter loss) or occlusion; (4) patients who were discharged early (within 3 days after admission); and (5) incomplete clinical data (e.g., lack of continuous neurological assessment). This study protocol was approved by the Ethics Committee of Dongguan People’s Hospital. Consent from the patients was waived because of the retrospective design of the study, which fulfilled the criteria for minimal risk to the patients.

### Collection of clinical data

Demographic characteristics (age and sex) and clinical characteristics, including vascular risk factors (e.g., hypertension, diabetes mellitus, hyperlipidemia, ischemic heart disease, frequent consumption of alcohol, and smoking history), previous stroke history, and treatment information, were collected from patients’ medical records. Neurological deficits caused by AIS were assessed using the National Institute of Health Stroke Scale (NIHSS) score recorded from the medical records. All patients received guideline-based treatments after admission.

### Definition of END

In our stroke unit, patients with AIS received a daily NIHSS assessment until 72 h after admission. The definition of END in our study refers to neurological deterioration with an increase in the NIHSS score ≥ 2 points in the first 72 h after admission [17, 18].

### MRI analysis

Brain MRI, including T1-weighted imaging, T2-weighted imaging, fluid-attenuated inversion recovery (FLAIR), DWI, SWI, and three-dimensional time-of-flight magnetic resonance angiography (3D-TOF-MRA) was performed for each patient using a 3.0 T system (Skyra, Siemens Medical, Germany) within 24 h after admission. Axial spin-echo T1 (TR/TE/excitation = 1500/11/1, FOV = 220 mm, slice thickness/gap = 4 mm/1.2 mm, matrix = 320 × 320, time of acquisition = 1 min 26 s) and turbo spin-echo T2 (TR/TE/ excitation = 4720/96/2, turbo factor 15, FOV = 220 mm, slice thickness/gap = 4 mm/1.2 mm, matrix of 512 × 512, time of acquisition = 1 min 50 s) images were also acquired. Coronal position FLAIR (TR/TE/excitation = 9000/84/1, FOV = 230 mm, slice thickness/gap = 5 mm/1.5 mm, matrix 320 × 320, time of acquisition = 1 min 50 s) and DWI spin-echo planar imaging (EPI) (TR/TE/excitation = 4640/67/1, matrix = 192 × 192, FOV = 230 mm, slice thickness/gap = 4 mm/1.2 mm, EPI factor = 91, acquisition time = 1 min 44 s) sequences with three orthogonally applied gradients were used, with b values of 0 and 1000. SWI (TR/TE/excitation = 27/20/1, FOV = 220 mm, slice thickness/gap = 3 mm/0.6 mm, matrix 256 × 256, time of acquisition = 2 min 28 s) and 3D-TOF-MRA (TR/TE/excitation = 21/3.42/1, FOV = 200 mm, slice thickness/gap = 0.7 mm/− 0.14 mm, matrix 384 × 384, time of acquisition = 3 min 36 s) were also conducted.

**Fig. 1:**
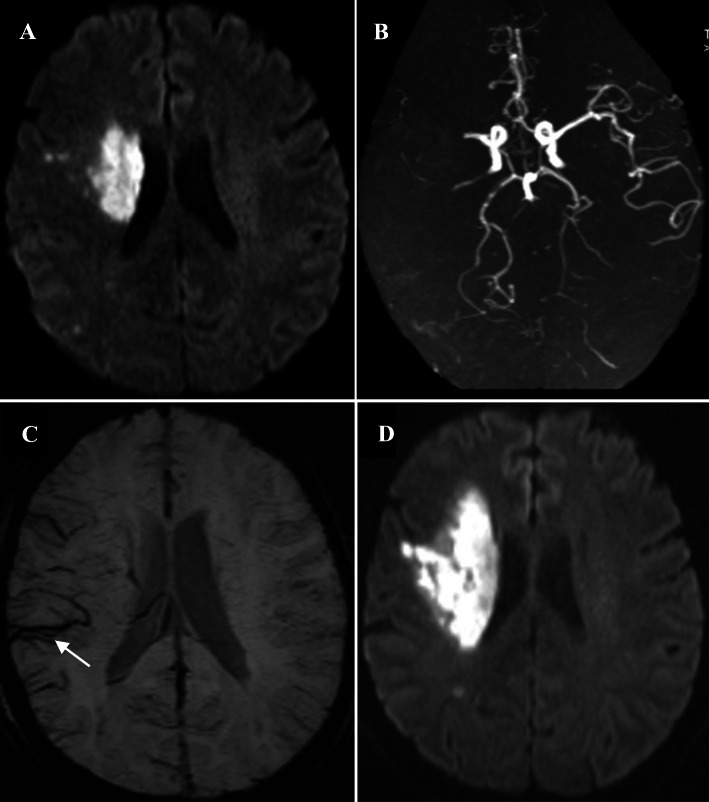
**a** Diffusion weighted imaging (DWI) in the first 24 h after admission: acute infarct in the right basal ganglia in one acute ischemic stroke patient with a National Institutes of Health Stroke Scale (NIHSS) score of 6 at admission. **b** Magnetic resonance angiography: occlusion in the right middle cerebral artery (MCA). **c** Susceptibility weighted imaging: asymmetrically prominent cortical vein sign in the right MCA territory (arrow). **d** Repeat DWI after early neurological deterioration (END), (Day 3 and the NIHSS score increased to 14): enlargement of the infarction

An experienced neuroradiologist and a trained neurologist (WF and YKC), who were blinded to the patients’ clinical information, independently assessed the MRI variables as well as the APCVS as follows:

1. Infarct. The location, number, and volume of acute infarcts were examined on DWI and the total volume was calculated by multiplying the total area by the sum of the slice thickness and the gap.
2. White matter lesions. The severity of white matter lesions was graded using the four-point scale of Fazekas et al. [19] Deep white matter hyperintensities and periventricular hyperintensities were scored.
3. Intracranial arterial stenosis or occlusion. This was assessed using the WASID Criteria [16]. Symptomatic severe intracranial arterial stenosis was defined as a stenosis ≥70% that affected the ICA or the M1 segment of the MCA ipsilateral to the infarction. Intracranial large artery occlusion was defined as signal loss of distal blood flow.
4. Hemorrhagic transformation. This was confirmed on the basis of signal changes on T1WI, T2WI, and SWI.
5. APCVS. This was the result of an internal comparison of vein signals between the two hemispheres. The APCVS was defined as more and/or a larger size of cortical veins with greater signal loss on the side with SIASO than on the contralateral size without SIASO in the minimum-intensity projection of SWI (Fig. 1) [10]. Ten cases were randomly selected to test the inter-rater and intra-rater agreement, and the results indicated good agreement (inter-rater kappa 0.82; intra-rater kappa 0.86).

### Statistical analysis

Statistical analyses were conducted using SPSS for Windows (v.19.0, SPSS Inc., Chicago, IL, USA). All patients were divided into two groups, the END (+) and END (−) groups. Differences between the groups with and without END were analyzed using Student’s *t*-test (for continuous variables), or the chi-squared test or Mann–Whitney *U* test (for categorical variables). Variables with *P* < 0.05 in the univariate analysis were included as independent variables in the multivariate logistic regression analysis to predict END. Statistical significance was set at *P* < 0.05 (two-sided).

## Results

During the study period, 1156 patients with AIS were admitted. Among them, 166 patients had symptomatic SIASO on the side ipsilateral to the acute infarction. We excluded 35 patients receiving intravenous thrombolysis (median NIHSS score: 7.5), 6 patients undergoing thrombectomy (median NIHSS score: 15.5), 10 patients without SWI, and 6 patients without complete clinical data (lack of NIHSS score within 72 h after stroke). Thus, a total of 109 patients (80 men and 29 women) were eventually included in this study, with a mean age of 64.2 (standard deviation, 11.8) years. Compared with the 57 excluded patients with SIASO, the included patients did not differ in age, sex, and NIHSS score at admission (*P* > 0.05). Of these patients, 30 (27.5%) developed END during the initial 72 h after admission despite standard treatment. Sixty (55%) patients presented with an APCVS on SWI.

### Relationship between APCVS and END

The APCVS was present in 24 (80%) patients in the END (+) group, whereas it was found in 36 (45.6%) patients in the END (−) group (*P* = 0.001). However, patients with an APCVS were more likely to have END than those without an APCVS (40.0% vs. 12.2%, *P* = 0.001). Additionally, compared with the END (−) group, the END (+) group had a larger infarct volume on DWI and was more likely to have a previous stroke history (Table 1). After adjusting for infarct volume on DWI and previous stroke history, the multivariate logistic analysis indicated that the APCVS remained a significant predictor of END [OR 4.349, 95% CI = 1.580–11.970, *P* = 0.004] (Table 2).

**Table 1:** Clinical characteristics of the sample and comparisons of variables between patients with and without END

	The whole sample *N* = 109	END (+) *N* = 30	END (−) *N* = 79	P
*Clinical variables*				
Age^*^	64.25 ± 11.78	66.4 ± 11.43	64.43 ± 11.88	0.242
Male n(%)+	80 (73.4%)	22 (73.3%)	58 (73.4%)	0.993
Hypertension n(%)+	81 (73.2%)	22 (73.3%)	59 (74.7%)	0.885
Diabetes melitus n(%)+	40 (36.7%)	14 (46.7%)	26 (32.9%)	0.183
Hyperlipidemia, n(%)+	40 (36.7%)	9 (30%)	31 (39.2%)	0.505
Ischemic heart disease, n(%)^¶^	7 (6.4%)	1 (3.3%)	6 (7.6%)	0.671
Atrial fibrillation n(%)^¶^	12 (11.0%)	2 (6.7%)	10 (12.7%)	0.505
Previous stroke n(%)^¶^	27 (24.8%)	3 (10%)	24 (30.4%)	0.028
Frequent consumption of alcohol n(%)^¶^	11 (10.1%)	2 (6.7%)	9 (11.4%)	0.37
Smoking n(%)+	53 (48.6%)	12 (40%)	41 (51.9%)	0.267
NIHSS score at admission^§^	4 (2–8)	4 (2–7.25)	5 (2–8)	0.379
SBP at admission*	154.46 ± 21.88	158.6 ± 19.9	152.9 ± 22.5	0.221
DBP at admission*	89.7 ± 13.7	90.0 ± 15.0	89.6 ± 12.3	0.875
Prestroke usage of antiplatelet agents, n(%)	17 (15.6%)	2 (6.7%)	15 (19.0%)	0.146
Prestroke usage of statins, n (%)	14 (12.8%)	2 (6.7%)	12 (15.2%)	0.342
Prestroke usage of antihypertensive agents, n (%)	31 (28.4%)	9 (30%)	22 (27.8%)	0.824
Prestroke usage of glucose lowering agents, n (%)	14 (13.4%)	6 (20%)	8 (10.1%)	0.169
Prestroke usage of anticoagulants, n (%)	3 (2.7%)	0	3 (3.7%)	0.560
Dual-antiplatelet agents after admission, n (%)	41 (37.6%)	13 (43.3%)	28 (35.4%)	0.448
*MRI variables*				
DWI-infarct volume (mm^3^)^§^	10.3 (2.3–19.7)	14.2 (6.6–21.9)	7.7 (1.6–18.2)	0.037
Hemorrhagic transformation n(%)^¶^	13 (11.9%)	3 (10%)	10 (12.7%)	1.000
WMHs^§^	2 (0–2.0)	2 (0–2.25)	2 (0–2.0)	0.847
APCVS n(%)	60 (55%)	24 (80%)	36 (45.6%)	0.001

*END* Early Neurological Deterioration, *NIHSS* National Institutes of Health Stroke Scale, *SBP* Systolic Blood Pressure, *DBP* Diastolic Blood Pressure, *DWI* Diffusion Weighted Imaging, *WMHs* White Matter Hyperintensities, *APCVS* Asymmetrically Prominent Cortical Vein Sign

*represents mean±SD, t-test; +represents n%, chi-square test; ¶ represents n%, Fisher exact test; § represents median (25Q-75Q), Mann-Whiteney U test

**Table 2:** Multivariate logistic regression of risk factors for poor outcome

Variables	β	OR(95%C.I.)	P
DWI-infarct volume (mm^3^)	−0.005	0.995 (0.997–1.014)	0.615
Previous stroke	−1.196	0.302 (0.080–1.136)	0.077
APCVS	1.470	4.349 (1.580–11.970)	0.004

*APCVS* Asymmetrically Prominent Cortical Vein Sign

## Discussion

In this study, we found that in AIS patients with SIASO, presence of the APCVS predicted a higher risk of END, which suggests that evaluation of perfusion status with SWI is applicable and helpful for prediction of END. Because of its lack of requirement for a contrast agent and high visibility, the APCVS might be a useful functional imaging marker for END in clinical practice.

END is an important problem in AIS because it is correlated with longer hospitalization and poor prognosis [20–22]. Although several serum biomarkers [23–26], vascular factors [27–31] and neuroimaging parameters [32, 33] have been reported to be potential predictors of END, persistent large artery occlusion has been recognized as a major independent risk factor for END. However, many patients with severe large artery stenosis or occlusion have neither a large infarction nor END because of good collateral circulation, and they may not have significant hypoperfusion. In patients with hypoperfusion, the cortical vein presents with a magnetic susceptibility effect that is increased on SWI, which results from the increased deoxyhemoglobin concentration. Several studies have shown that the APCVS is present in AIS patients with unilateral artery stenosis or occlusion [10, 13, 34, 35], and a systematic review reported that the cumulative prevalence of APCVS on SWI in these studies was 81% (range 34–100%) [36]. The presence and range of the APCVS in patients with intracranial arterial occlusion has been shown to coincide with hypoperfusion confirmed by magnetic resonance perfusion-weighted imaging [37].

In practice, clinicians are highly concerned about END. However, few studies have examined the relationship between the APCVS and END. Sun et al. [15] found that in AIS patients with MCA territory, the APCVS might be considered a neuroimaging predictor for END. In their study, the APCVS was present in 39 patients, and 37 (94.9%) of these patients had ipsilateral ICA/MCA stenosis or occlusion. However, the authors did not exclude the effects of ipsilateral ICA/MCA stenosis or occlusion on END because persistent occlusion of the large arteries has been recognized as a major independent risk factor for END. Thus, ipsilateral ICA/MCA stenosis or occlusion should be considered in the analysis of these patients. Considering this confounding factor, our research was focused on SIASO patients, and AIS patients with contralateral ICA or MCA stenosis (more than 50% diameter loss) or occlusion were excluded because the APCVS results were from a comparison within an individual [38]. Another study reported [39] that a peripheral APCVS was positively correlated with the degree of MCA stenosis, and fewer peripheral APCVSs may suggest a favorable outcome of unilateral MCA infarction at the 3-month follow-up. However, they did not elaborate on the relationship between the APCVS and AIS patients in the acute phase.

Our study found that patients with an APCVS had a higher risk for END (OR = 4.3) on the basis of symptomatic SIASO, even after adjusting for possible confounders, which suggests that the APCVS is a reliable imaging marker for END.

There are several limitations to this study. First, the retrospective design could not confirm a causal relationship between APCVS and END. We did not follow up on patients after their admission and did not investigate 3-month or long-term outcomes. Future studies will therefore explore whether the APCVS can predict the recurrence of ischemic stroke or intracranial hemorrhage. Second, the APCVS was not evaluated with a satisfactory quantitative measurement. We did not measure the medullary vein sign, another SWI marker of hypoperfusion, in this study. In the future, autonomic computed assessment tools are therefore warranted to achieve a more accurate and reliable result. Third, we were unable to classify the exact reason for END and for patients with fluctuating NIHSS scores in this retrospective study. Fourth, it should be noted that the findings in this study are limited to the target subjects set by the inclusion and exclusion criteria and are not applicable to patients with bilateral SIASO.

## Conclusion

We demonstrated that the APCVS is a strong neuroimaging marker for END in AIS patients with ipsilateral SIASO, which suggests the importance of evaluating perfusion status. SWI should be a routine MRI sequence in patients with AIS because of its capacity to safely and conveniently assess perfusion status.

## Data Availability

The datasets generated and/or analyzed during the current study are not publicly available because they are personal data, but they are available from the corresponding author on reasonable request.

## Abbreviations

SWI: Susceptibility weighted imaging
END: Early neurological deterioration
SIASO: Severe intracranial arterial stenosis or occlusion
NIHSS: National Institutes of Health Stroke Scale
AIS: Acute ischemic stroke
DWI: Diffusion weighted imaging
ICA: Internal carotid artery
MCA: Middle cerebral artery
APCVS: Asymmetrical prominent cortical vein sign
WASID: Warfarin Aspirin Symptomatic Intracranial Disease
